# The mediating role of anxiety between negative feelings and depression among students with congenital physical disabilities

**DOI:** 10.1371/journal.pone.0281430

**Published:** 2023-03-02

**Authors:** Marie Paule Uwimbabazi, Jean d’Amour Muziki, Assumpta Muhayisa, Thaoussi Uwera, Jean Mutabaruka

**Affiliations:** 1 Department of Clinical Psychology, College of Medicine, and Health Sciences, University of Rwanda, Kigali, Rwanda; 2 National Child Development Agency (NCD-Rwanda), Rwanda; 3 Department of Health Informatics, College of Medicine and Health Sciences of University of Rwanda, Kigali, Rwanda; Hangzhou Normal University, CHINA

## Abstract

Congenital physical disability is associated with various psychological challenges, including negative feelings, anxiety, and stress. These challenges will, in turn, predict significant negative emotional well-being among students with congenital physical disabilities, but the mechanisms of these effects are not well known. This study examined whether Negative Emotional Wellbeing Anxiety (NEWA) would mediate the effect of Negative Feelings (NF) and Negative Emotional Wellbeing Depression (NEWD) among students with congenital physical disabilities. Forty-six students with congenital physical disabilities (*mean age*: *20 years*, *SD = 2*.*05*; *45*.*65% females*) completed self-rating measures that included sociodemographic variables (age and sex), emotional state for Children to assess negative feelings, and an emotional distress protocol to assess NEWA and NEWD. Results show that NF was positively correlated with NEWA (*r =* .*69*, *p <* .*001*) and NEWD (*r =* .*69*, *p <* .*001*), and NEWA was positively correlated with NEWD (*r =* .*86*, *p <* .*001*). Findings further reported that NEWA significantly mediated the positive relationship between NF and NEWD (a*b = .*37*, *Bootstrap CI95 =* .*23 and* .*52*) [*Sobel test statistic of 4*.*82* (*p <* .*001*)] among students with congenital physical disabilities. The results highlight the importance of screening students with congenital physical disabilities for common psychological challenges and providing suitable interventions.

## Introduction

Congenital physical disabilities are defined as any loss or abnormality of a physiological or anatomical structure or function present at birth due to various causes such as prematurity, perinatal anoxia, maternal malnutrition, rubella, toxoplasmosis, birth trauma, radiation exposure, use of drugs, metabolic, and other unknown causes [1]. They exist in many different forms, like amputation or absence of a limb, cerebral palsy, hemiparesis, hemiplegia, malformed limbs, monoparesis, monoplegia, paraparesis, paraplegia, quadriplegia, spina bifida, tetraparesis, triparesia, and triplegia [1]. It is well known that the nature of acquisition and the sociological impact of congenital physical disabilities have much more negative effects than that of acquired or progressive disabilities [2].

Congenital, acquired or progressive disability is a big concern for both the public health and social sectors [3]. It was revealed that difficulties in functioning as a result of psychological, intellectual, or physical impairment were experienced by almost 200 million people out of 1 billion by 2012 with a significant type of disability [4]. Besides, most available studies report that adolescents or children with different types of disabilities experience negative feelings depending on environmental factors such as people’s attitudes and reactions to their deficiency [5], diminished interactions with peers and classmates, a lack of enough friends at school, and limited availability of companions [6, 7]. Individuals with negative feelings can experience anger, disgust, fear, gloom, guilt, loneliness, misery, nervousness, sadness, scare, shame, and upset [8].

Taking account of the foregoing, it has been reported that rejection, exclusion, and withdrawal by peers hinder their socialization by reducing cultural opportunities [6, 7], and lead to specific negative feelings such as isolation, envious/jealous, embarrassment for oneself, and concerns about bothering others [9, 10]. According to similar studies, children and adolescents with acquired or congenital physical disabilities generally feel less socially accepted and physically unable than their biological peers [11, 12]. It has also been suggested that depriving them of social activities can lead to negative feelings of low self-confidence and self-esteem, worries about their disabilities, and a lack of skills [13, 14]. These children can also experience negative feelings of depression and unhappiness [6, 14]. In fact, it is irrevocably admitted that being a person with a disability is a risk factor that leads to a high level of negative emotional well-being and depression [15] which causes adolescents with a congenital physical disability to feel like a failure, worthless, helpless, sad, depressed, hopeless, and unhappy [16]. However, effectively treating symptoms of negative emotional well-being such as depression can repair negative feelings [17].

A longitudinal study showed that individuals with predominant negative feelings are overall at higher risk of experiencing negative emotions such as anxiety, psychological distress, and dissatisfaction with life, and they tend to focus on unpleasant aspects of their personal life, surrounding people, the world, and the future, and will frequently recall negative life events [18]. The most common negative emotion in our present study is anxiety due to its established relationship with both negative feelings and depression [19–21]. This negative emotional well-being anxiety is characterized by feeling fearful, anxious, worried, nervous, uneasy, tense, and finding it difficult to focus on anything other than anxiety [16]

### Rationale

Evidence shows that the broader capacity of adolescents with congenital, acquired, or progressive disabilities is neglected while their aspirations are limited [22]. This population faces isolation in the virtual and real worlds and disadvantages resulting from social and gender discrimination [23]. It is challenging for them to access quality health care and education, learn social skills, and socialize with peers [23]. These challenges heighten the stigma and psychological problems encountered by adolescents with disabilities and their families [24]. Regarding what is not known, there has been very little research on adolescents with disabilities as a more vulnerable group in developing countries, and what is known about adolescents with disabilities in developed countries focused on their formal educational systems and programs for making the transition from school to the workforce [25]. Besides, the call for further study on the various issues that have an impact on the lives of adolescents with disabilities made by UNICEF in 1999 in its global survey on adolescents is still unanswered [26], particularly in developing countries like Rwanda where there is still a lack of research on psychological problems experienced by persons with disabilities, especially children, and adolescents with congenital physical disabilities [24].

Though studies have shown that negative feelings [5], anxiety [27], and depression [28] are common in children with disabilities, studies assessing the relationships between negative feelings, anxiety, and depression are still lacking in the sample of students with congenital physical disabilities. One study conducted on adult people from the general population using mediation analyses revealed that people with more significant revenge-related negative feelings were at a higher risk of experiencing both anxiety and depression symptoms [29]. Therefore, it is likely that students with congenital physical disabilities who have experienced negative feelings will be more at risk of developing symptoms of depression. Such patterns of anxiety related to depressive symptoms and negative feelings may be an important indicator of a plausible role as a mediator.

In a nutshell, studies on negative feelings, anxiety, and depression have been conducted. However, no research has examined the mediation role of Negative Emotional Wellbeing Anxiety (NEWA) in the relationship between Negative Feelings (NF) and Negative Emotional Wellbeing Depression (NEWD) among students with congenital physical disabilities. Based on the previous findings, this study intends to fill the gap by examining comprehensively the relationship between NF (independent variable), NEWA (mediating variable), and NEWD (dependent variables) in students with congenital physical disabilities from HVP-GS Gatagara-Huye in Rwanda. This paper aims to provide theoretical guidance to alleviate and effectively prevent the sense of negative emotional well-being among students with congenital physical disabilities and reduce their level of negative feelings. We suppose that increased NEWA may heighten the psychological distress of students with congenital physical disabilities who report both higher rates of NF and NEWD. Consistently, researchers reported that NF is highly associated with NEWD [30, 31] while NEWA is highly associated with a higher rate of NEWD [32, 33]. Therefore, two hypotheses and one exploratory research question are formulated. First, we expect a higher score of NF to be associated with a higher NEWD score in our sample of students with congenital physical disabilities. Second, we hypothesize that a higher NEWA rate is associated with a higher rate of NEWD. The exploratory research question is whether and to what extent NEWA may mediate the relationship between NF and NEWD.

## Methods

### Study design, sample, and procedures

The study was institution-based with a cross-sectional study design. A sample composed of 46 students (21females and 25 males) was selected from only one inclusive school in Huye-Rwanda, «Home de la Vierge des Pauvres Gatagara» HVP-GATAGARA, between March and September 2017. The total number of students in this school was 117 females and 252 males between the ages of 16 and 23. Those with congenital physical disabilities were 54 altogether (26 females and 28 males). They had the following congenital physical disabilities: amputation, spina bifida, cerebral palsy, and malformed limbs. However, those eligible were 46 students and were registered in lower secondary school. This convenience sample falls in the acceptable range for adolescence (10–24 years old), which is now an informative period for adolescent development and popular understanding of this life phase [34]. Participants were recruited in close collaboration with the director of the institution. Inclusion criteria were students with one or more forms of congenital physical disabilities such as amputation, spina bifida, cerebral palsy, and malformed limbs. They were also between 16 and 23 years old, willing to participate voluntarily in the study, and able to read and respond to the self-report questions on the research instruments. However, students with congenital physical disabilities who were distressed by the questionnaires, those with communication problems, severe mental disturbance, or other severe illness that would affect their judgment were excluded from the study.

Ethical approval was obtained from the Institutional Review Board of the University of Rwanda, CMHS (IRB-CMHS). Once informed of the research objectives and ethical guidelines, those aged 18 years and above provided written consent to participate in the study while we obtained consent from parents or guardians of the minors. Two research assistants were trained to provide emotional support to the participants with emotional challenges and later refer them to the school counselor. The participants were assured that it was their right to withdraw from the study if they did not want to participate or if they changed their mind. Research questionnaires were granted for non-commercial applications and were then translated into Kinyarwanda to facilitate participants’ responses to the questionnaires and back-translated respectively by three psychologists who have proficiency in Kinyarwanda, French, and English.

### Measurements

The first validated research instrument for this study is the Emotional State for Children. It is a 30-item self-report questionnaire that measures positive and negative feelings for children and adolescents [8]. According to the recent inclusive definition of adolescence, this period lasts from the ages of 10–24 for providing suitable services associated with a child or adolescent’s needs [34]. Therefore, study participants aged between 10–24 years old were assessed using the Emotional State for Children. This scale assessed fifteen negative feelings (NF) of children or adolescents on a five Likert rating scale: not much or not at all, a little, some, quite a bit, and a lot. The participants were asked to report the frequency of their mood during the “past few weeks.” In our sample, the internal consistency was .92 for negative feelings.

The Emotional Distress protocol is another validated research instrument for the current study. It is a 23-item self-report questionnaire that assesses Negative Emotional Wellbeing such as depression (NEWD) (e.g., I felt like a failure) and anxiety (NEWA) (e.g., I felt fearful) [16]. The participants reported their emotional distress in the past 7 days on a 5-point Likert-type scale ranging from 0 (never) to 4 (always) and the extent to which they felt each item for each subscale. The Cronbach’s alpha in our sample was .94 for the depression subscale and .89 for the anxiety subscale.

### Statistical analysis

Data were analysed using the Statistical Package for the Social Sciences (SPSS) version 24. Then, multiple regression analyses were performed to test the function of the mediator (NEWA) in the causal sequence by following instructions established by Baron and Kenny [35]. Finally, Hayes SPSS Process Macro with a bootstrapping technique was employed to determine with the more precision indirect effect of the moderating variable (NEWA) on the relationship between the independent (NF) and dependent variable (NEWD) [36]. As stated by Baron and Kenny, four conditions have to be fulfilled to confirm the indirect effect of the moderator [35]. First, the independent variable (NF) should be a statistically significant predictor of the dependent variable (NEWD). After that, the independent variable (NF) should be a statistically significant predictor of the mediator (NEWA). Then, the mediator (NEWA) should be a statistically significant predictor of the dependent variable (NEWD) while controlling for the effect of the independent variable (NF). Finally, there is a full mediation when NF no longer statistically significantly affects NEWD after controlling for NEWA, while partial mediation occurs when the effect of NF on NEWD is still statistically significant but reduced. PROCESS version 4.0 was used to overcome the problem of small samples [36]. In small to moderate samples, this bootstrapping digitalized tool was found to be reliable for testing the indirect, partial, and total effects of an independent variable on the dependent variable [36].

## Results

### Descriptive analysis

The study sample was composed of 46 students (mean age: M = 20 years; SD = 2.05; 21 [45.65%] females, and 25 [54.34%] males). The variables of interest were NF, NEWA, and NEWD. Table 1 presents the descriptive statistics of those study variables. This table shows that the participants’ mean NF score was 35.98 (SD = 13.36), the mean score for NEWA was 19.30 (SD = 7.38), and the mean NEWD score was 19.36 (SD = 9.34). To determine whether the scale’s distribution showed a normal distribution, the kurtosis and skewness were examined. All the study scales were found in the normal range, which assumes that skewness and kurtosis values should fall between *-*2 and +2 [37]. In addition, bivariate correlation analyses showed that NF was positively correlated with NEWA (r = .69, p < 0.001) and NEWD (r = .69, p < 0.001), and NEWD was positively correlated with NEWA (r = .86, p < 0.001). These findings confirm a plausible mediating effect.

**Table 1:** Descriptive statistics for NF, NEWA and NEWD (N = 47).

	M	SD	Skewness	Kurtosis	NF	NEWA	NEWD
**NF**	35.98	13.36	0.648	-0.371	1		
**NEWA**	19.30	7.38	0.243	-0.978	.699**	1	
**NEWD**	19.37	9.34	0.484	-0.745	.693**	.867**	1

**. Correlation is significant at the 0.01 level (2-tailed).

Mean (M), Standard deviation (SD), Negative feelings (NF), Negative emotional wellbeing anxiety (NEWA). Negative emotional wellbeing depression (NEWD).

### Mediations

**Fig 1:**
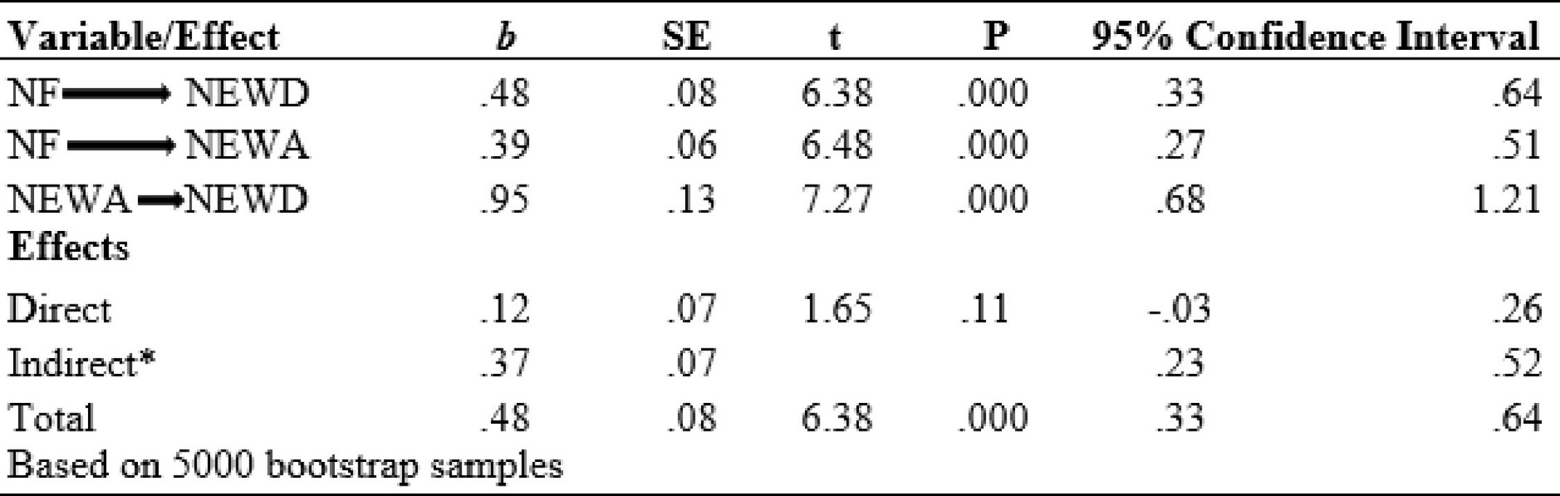
Mediation analysis.

**Fig 2:**
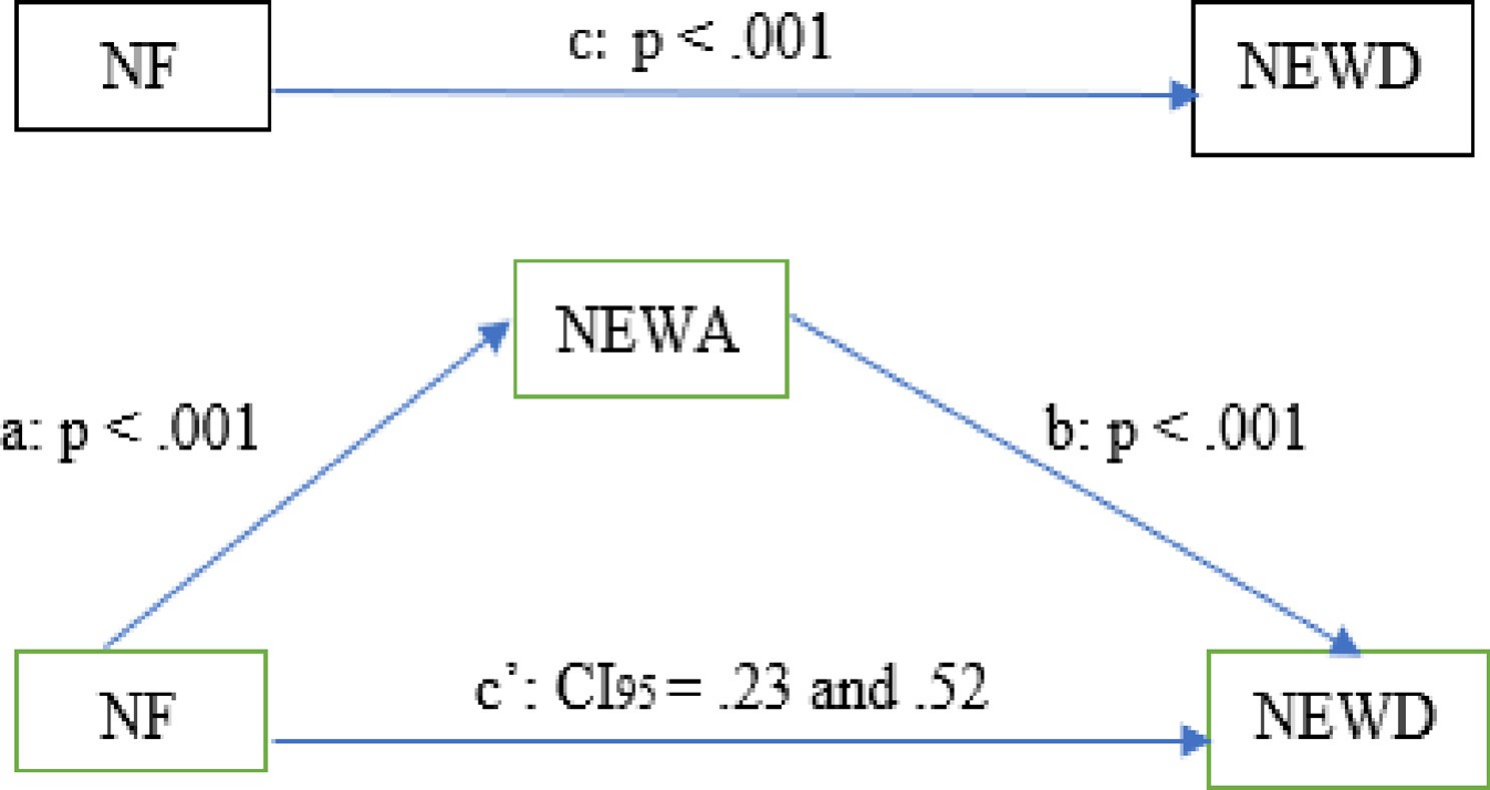
Model of the mediating role of NEWA between NF and NEWD.

The results of the regression analysis show that NF (independent variable) was a significant predictor of NEWD (dependent variable) (*b* = .48, t = 6.38, p < .001) and NEWA (mediator) (*b* = .39, t = 6.48, p < .001). NEWA was also a significant predictor of NEWD (*b* = .95, t = 7.27, p < .001). Next, while controlling for the mediator (NEWA), the results from regression analysis show that NF was not a significant predictor of NEWD (*b* = .12, t = 1.65, p > .05). These results prove that there was no direct effect of NF. However, based on 5000 bootstrap samples, the indirect effect results demonstrate a strong indirect positive relationship between NF and NEWD mediated by NEWA (a*b = .37, Bootstrap CI95 = .23and .52). According to the results of this bootstrap method, both the lower and upper bounds of the CI do not contain 0, and this confirms the significance of the mediating effect. The NEWA (mediator) accounted for approximately 77% of the total effect on NEWD [PM = (.37)/ (.48)]. The results of the mediation analysis are presented in Figs 1 and 2. Additionally, the presence of indirect mediation was verified by the Sobel test statistic of 4.82 (p < .001)

## Discussion

The main objective of this study was to investigate whether the relationships between Negative Feelings (NF) and Negative Emotional Well-being Depression (NEWD) could be accounted for by Students with congenital physical disabilities’ Negative Emotional Well-being Anxiety (NEWA).

First, the positive correlation between NF and NEWA is consistent with earlier studies. For instance, Craske showed that negative feelings are at the core of different psychopathologies, especially depression and anxiety [38]. Fernandez & Kerns found that negative feelings are primarily anger, fear, and sadness, which are key clinical symptoms of anxiety [39]. However, experiencing negative feelings is a risk factor for a poor quality of life and lower resilience among adolescents with congenital physical disabilities [40]. Second, findings revealed that NEWA was significantly and positively associated with NEWD among students with congenital physical disabilities. That is, a higher level of NEWA symptoms among the participants is associated with increased NEWD. Previous studies observed the positive relationship between NEWD and NEWA among children and adolescents with different forms of disability [28, 41] Other studies have demonstrated that NEWA and NEWD co-occur, symptoms of NEWA usually precede the onset of the symptoms of NEWD, and NEWA is a risk factor for NEWD among various samples, including children and adolescents with disability [42, 43].

Third, NF was also found to be significantly and positively associated with NEWD among students with congenital physical disabilities. These findings are consistent with a recent study that showed that negative feelings play a significant role in the development of different psychopathologies, including depression and anxiety, and are considered key symptoms in psychodiagnostic assessment for further treatment [44]. Students with congenital physical disabilities may be at higher risk of experiencing negative feelings due to academic responsibilities and day-to-day challenges such as pressure to perform well at school, limited skills and abilities to use school resources, negative attitudes and reactions, lack of companions, and feeling physically unable compared to others. The situation can be worse if there are few or no community-inclusive programs or political structures for the inclusion of people with disabilities. Therefore, a lack of support at different levels in society can contribute to NF among children with congenital physical disabilities. The significant positive relationship between NF and NEWD is consistent with the theory stating that depression is characterized by higher symptoms of negative affect and a lower level of positive affect [30].

Finally, the indirect effect of NF on NEWD among children with congenital physical disability identified NEWA as a significant mediator in the relationship between NF and NEWD. That is, higher symptoms of NF were positively and significantly associated with higher symptoms of NEWD through increased NEWA. In this prediction, the NF predicted an increased NEWA level, which is in line with what has been observed in the previous studies [45, 46], and the increased level of NEWA predicted a higher level of NEWD [33, 47] Therefore, increased levels of NEWA are one of the plausible mechanisms underlying the negative effect of NF on NEWD among student with congenital physical disability.

### Strength and implications for policy, practice, and research

The main strength of this study lies in its being conducted in one of the sub-Saharan countries where there is a high mental health burden and scarce studies, especially on vulnerable groups like people with congenital physical disabilities. With regard to the study implications, study findings suggest that more rehabilitation strategies should be introduced by policymakers and their partners to make students with congenital physical disability part of their respective schools and community activities, regardless of their strengths and weaknesses, to help them meet their standard of psychological wellbeing and mitigate the effects of mental health-related problems.

In addition, this study showed that students with congenital physical disabilities suffer from negative feelings that increase their level of negative emotional wellbeing, such as anxiety and depression. These findings imply that NEWD and its predictors (NF and NEWA) among students with congenital physical disabilities should be assessed earlier by suitable practitioners for further management. As a group, people with congenital physical disabilities are at risk for poor mental health. Therefore, the findings from this study revealed that future researchers should focus on testing psychological interventions that can replace the negative image with a positive one in the group of people who have congenital physical disabilities.

### Limitations

First, students with congenital physical disabilities who were distressed by the questionnaires were excluded from the study. However, their psychological distress towards the questionnaire was addressed in a clinical setting. Second, the study was cross-sectional, the sample was small, and results were almost certainly influenced by specific characteristics of these students with congenital physical disabilities from HVP-GATAGARA/Huye and might very well fail to generalise to other students with congenital physical disabilities in other countries. Therefore, a similar study with a larger sample size and proper probabilistic sampling may improve conclusions and inferences made by basing them on the larger sample. Despite this limitation, this is one of the first studies that explores the psychological aspects of students with congenital physical disabilities in Rwanda and provides an important basis for future researchers. A final limitation is methodological. The reason is that the tools that were used in this study were not validated for the Rwandan culture. So, we were required to provide more explanations to the participants to make the items of the questionnaires more understandable. Despite this challenge, Cronbach’s alpha did show that the internal consistencies of the tools used in this study were in an acceptable range.

### Conclusion

Based on the study findings, it is concluded that students with congenital physical disabilities experience negative feelings that increase their level of negative emotional well-being. Thus, an assessment of common psychological challenges among students with congenital physical disabilities is needed for suitable interventions.

## Supporting information

S1 Dataset https://doi.org/10.6084/m9.figshare.19683306.v1. (SAV)
